# A hybrid lesion of intralobar sequestration with mixed features of CPAM type I and type II unmasked following SARS-CoV-2 infection: Case report and literature review

**DOI:** 10.1016/j.ijscr.2022.107336

**Published:** 2022-06-22

**Authors:** Dehua Wang, William B. Wheeler

**Affiliations:** a Department of Pathology and Laboratory Medicine, Children's Minnesota, Minneapolis, MN 55404, The United States of America; b Department of Pathology and Laboratory Medicine, Rady Children's Hospital UCSD, San Diego, CA 92123, The United States of America; c Respiratory & Critical Care Specialists, Children's Minnesota, Minneapolis, MN 55404, The United States of America

**Keywords:** Hybrid lesion, Intralobar sequestration, CPAM, Congenital cystic lung lesions, SARS-CoV-2, Case report

## Abstract

**Introduction:**

Hybrid lesions of intralobar sequestration (ILS) associated with congenital pulmonary airway malformation (CPAM) is rare and could be undetected by prenatal ultrasound. Some of the cases are discovered incidentally or following lung infection in late childhood or adulthood.

**Case presentation:**

17-year-old female developed chest pain, non-productive cough, low grade fever, and sore throat several weeks following SARS-CoV-2 infection. CT angiogram revealed a large lobulated cystic mass with celiac arterial supply in the posterior right lower lobe that was diagnostic for pulmonary sequestration. Gradually she recovered from all respiratory symptoms after a course of multiple antibiotic treatment for symptom relief. In order to prevent recurrent infection and malignancy, she underwent right lower lung mass resection approximately 3 months later.

**Discussion and conclusion:**

Pathological examination confirmed a hybrid lesion of ILS with mixed features of CPAM type I and type II. The hallmark morphological features of SARS-CoV-2 infection were not identified except for those of superimposed acute and chronic bronchopneumonia, abscesses formation and fibrosis within the lesion. This is the first case report of a hybrid lesion of ILS associated with CPAM type I and type II, unmasked following SARS-CoV-2 infection. By using the term of hybrid lesion to report this case is to efficiently correlate the terminology and nomenclature applied in the literature currently for multidisciplinary communication between radiology, pulmonary, surgery and pathology.

## Highlights

• Pulmonary sequestration with congenital pulmonary airway malformation type II is rare and discovered incidentally.
• This is the first case report of a hybrid lesion of intralobar sequestration with congenital pulmonary airway malformation type I and type II.
• The hybrid lesion was unmasked following SARS-CoV-2 infection.
• The term of hybrid lesion used to report this case is for effective multidisciplinary communication.

## 1 Introduction

Congenital cystic lung lesions (CCLL) are a heterogeneous group of rare clinical-pathological malformations. CPAM and pulmonary sequestration (PS) are the most common CCLL diagnosed prenatally. PS is a rare congenital anomaly with systemic arterial supply and non-functional lung tissue uncommunicated to the tracheobronchial tree. There are two distinguished types of PS. ILS is located within the visceral pleural of a normal lung lobe, and next to the normal functional lung parenchyma. Extralobar sequestration (ELS) has its own visceral pleural and is located either outside the functional lung lobe in the thoracic or outside the thoracic cavity. The frequency of ILS and ELS are approximately 75 % and 25 %, respectively [1]. A few retrospective pathological case series in fetus and infant have demonstrated PS commonly associated with CPAM type II [2]. However, some of the associated CPAM lesions in PS were not specified or subtyped in these case series [3]. The term of hybrid lesion has been used widely to describe PS associated with CPAM in the literature particulately in clinical and radiological articles after the terminology was introduced in 1997 [4]. The reported cases of hybrid lesions of PS in children or adults were mostly associated with CPAM type II and focused on unique clinical presentations and radiological features [5], [6], [7]. PS associated with CPAM type I has not been fully illustrated and described.

The epidemiological and economic impact of COVID-19 has been noted globally. Cystic lung disease in adults as a sequela of severe COVID-19 infection has been reported during pandemic. Only one case report of CCLL incidentally discovered in an adult during COVID-19 screening was published recently [8]. We describe a case of hybrid lesions of ILS associated with CPAM unmasked following SARS-CoV-2 infection in a teenage girl. This resulted in early surgical resection of her lesion to prevent progression of chronic pneumonia or malignancy. The peculiar presentation of this hybrid lesion of ILS associated with mixed features of CPAM type I and type II in a pediatric patient during COVID pandemic has not been reported to our knowledge. The case is reported using the SCARE 2020 criteria [9].

## 2 Case presentation

### 2.1 Clinical history

**Fig. 1:**
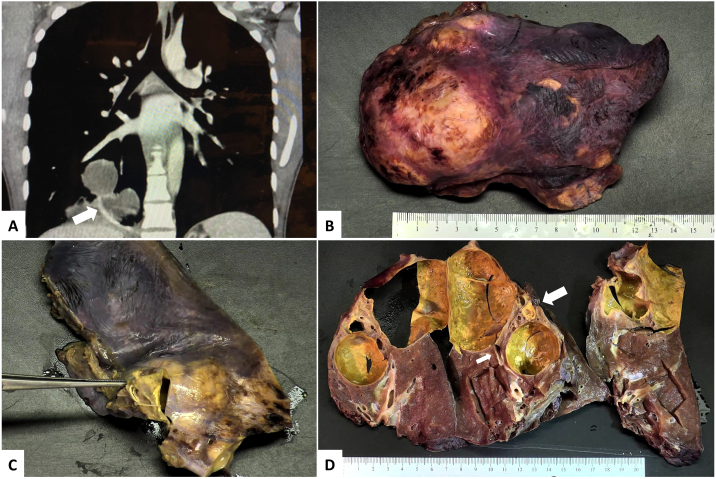
Intralobar pulmonary sequestration. Contrast CT (A) showing a lobulated cystic mass with celiac arterial blood supply (arrow); lobectomy specimen of right lower lobe (B) showing a bulging cystic mass; the large anomalous systemic celiac artery next to the forceps (C); the ill-defined cystic lesion (D) composed of two large cysts (4 cm and 9 cm) with round to irregular cystic cavity and prominent residual yellow precipitation after abundant purulent thick fluid empty out during serial sectioning, and multiple small cysts range from 0.1 cm to 0.3 cm (large arrow); one bronchus behind the large cyst (small arrow) to the peripheral upon dissection of the bronchus. (For interpretation of the references to colour in this figure legend, the reader is referred to the web version of this article.)

An asymptomatic 17-year-old female was screened for COVID-19 due to her part time employment in a nursing home after a family exposure. Her nasal RT-PCR study was positive for SARS-CoV-2, and she was quarantined for 2 weeks without illness. 5 weeks later she developed chest pain, non-productive cough, low grade fever, and sore throat. She had no prior history of asthma, allergies, chest illness. The family history was negative for genetic syndromes or chronic lung diseases. The chest x-ray revealed two nodular opacities in the right lower lobe (RLL). CT angiogram revealed the posterior RLL had a large lobulated cystic mass with celiac arterial supply that was diagnostic for pulmonary sequestration (Fig. 1A).

The differential diagnosis at this time included chronic pneumonia from SARS-CoV-2 infection, pulmonary malignancy, post-infectious bronchiectasis, and congenital lesion of the lung with secondary infection. Flexible bronchoscopy with bronchoalveolar lavage showed only erythema suggesting tracheobronchitis but negative viral, bacterial, and fungal cultures. A rapid viral PCR panel was positive for only rhinovirus, and negative for SARS-CoV-2. She was treated with ceftriaxone 2 g IM, cefdinir 300 mg po BID for 10 days, and azithromycin 500 mg po daily for 3 days.

Her fever and sore throat resolved within ten days, but a dry non-productive cough persisted. Due to concern for persistent/recurrent pneumonia or malignant transformation of her intralobar sequestration, she underwent thoracoscopic resection of her RLL by an experienced board certified pediatric surgeon at our large pulmonary referral center two and half months later. Her post-operative course was complicated by persistent right-sided pneumothorax responsive to autologous blood patch instillation at 5 days post procedure. She was discharged two days later. At her one-year follow-up she has a normal chest exam, normal pulmonary function, and minimal pleural thickening of the RLL on chest radiograph. Her exercise capacity is reportedly normal. She feels much relieved after having the congenital lesion removed, and is now attending university.

### 2.2 Pathological findings

**Fig. 2:**
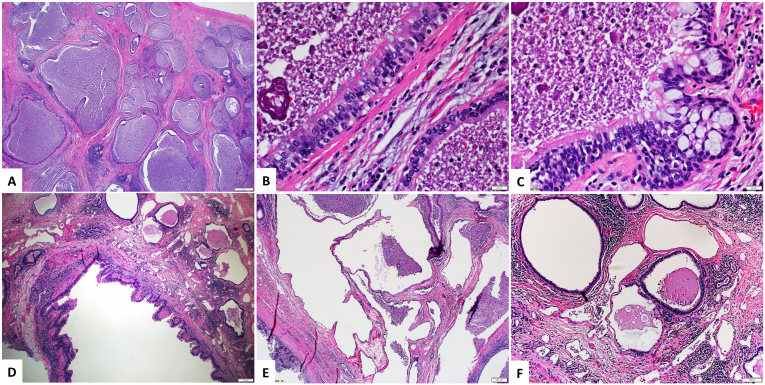
A hybrid lesion with features of CPAM type I and type II in intralobar sequestration. The variable sized cysts (A, 20×) filled with eosinophilic debris, mucin, and inflammatory cells and lined by ciliated bronchial and bronchiolar epithelial cells and thin fibromuscular wall (B, 400×); rare clusters of mucous cells (C, 400×); one cyst with epithelial papillary projections and prominent fibromuscular wall (D, 100×); the bronchial epithelium lined cysts connecting to the large alveolar–like spaces (E, 40×); occasional alveolar-like spaces intermingled with bronchiolar epithelium-lined cysts (F, 100×).

**Fig. 3:**
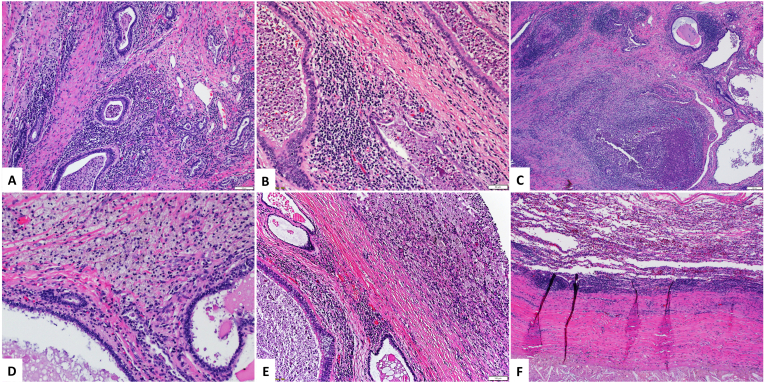
Superimposed infection and fibrosis within the hybrid lesion. Acute and chronic inflammation, fibrosis (A, 200×; B, 200×); ruptured cysts with abscesses formation (C, 40×); prominent muciphages accumulation (D, ×200); the wall of the large cyst replaced by marked fibrosis and precipitation of cystic content (E, 100×, right side); the large cyst compressing the adjacent alveoli (F, 40×).

**Fig. 4:**
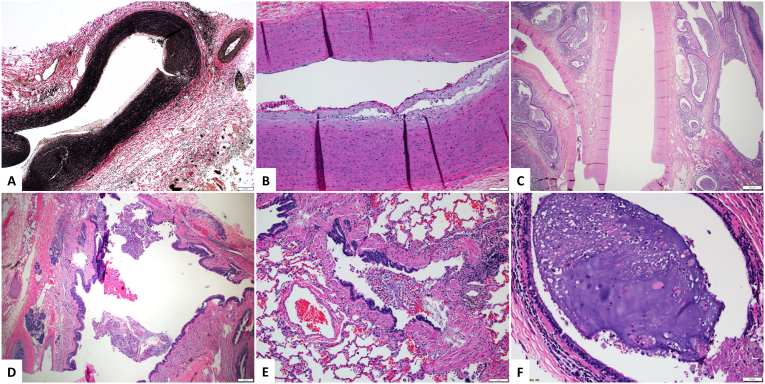
Hybrid lesion with the anomalous celiac artery and the adjacent functioning bronchial tree. Cross section of the celiac arterial showing elastic artery (A, elastic stain 20×) with intimal thickening and myxoid change (B, 100×); the celiac artery at insertion point ramifying (C, 100×); the adjacent hilar bronchus (D, ×40) and terminal bronchiole (E, ×100) without evidence of bronchial atresia but small amount of intraluminal mucin mixed with inflammatory cells and minimal chronic inflammation in the walls, in contrast to mucous plug in the small cyst within the lesion (F, 200×).

The RLL (Fig. 1B) showed a large cystic mass in the posterior lower portion of RLL. The anomalous celiac systemic artery was identified on the pleural surface of the mass (Fig. 1C). Serial sections of RLL along the bronchial tree from hilum to the mass showed an ill-defined multicystic lesion (Fig. 1D). Upon dissection of the bronchial tree, no mucous plugs were identified within the lumen of bronchial tree. The bronchial tree was not communicated to the cysts. Microscopic examination demonstrated mixed features of CPAM type I and type II (Fig. 2A-F). No cartilage plate was present. There was no immature mesenchyma. No cytological or architecture atypia or mitosis was identified. There was a prominent acute and chronic inflammation, abscesses formation and fibrosis within the lesion (Fig. 3A-F). No features of diffuse alveolar damage or thrombi were seen. The anomalous branches of celiac artery demonstrated an elastic artery with hypertensive-like changes (Fig. 4A-C). No atretic bronchi were identified (Fig. 4D-F).

## 3 Discussion and conclusion

CPAM/congenital cystic adamantoid malformation (CCAM), PS and related hybrid lesions, bronchogenic cyst, congenital lobar emphysema/congenital alveolar overinflation, polyalveolar lobe and isolated bronchial atresia are relatively common CCLL. Clinical impression and radiological study may not be able to distinguish most of them. Many diagnoses based on imaging have to be revised after pathological examination. CCLL has been subjected to many revisions and reclassifications. The terminology and classifications can be very confusing to clinicians and pathologists during clinical communication. CPAM is currently the broadly accepted term to replace the old term of CCAM, as cystic change is not always present [3]. Although the modified Stocker classification (5 subtypes) [10] has been popularly used in practice and literature reports, the basic pathogenetic mechanism of the classification has been facing challenges. Langston and other subsequent case series studies have identified bronchial atresia in the specimens of these cystic lung lesions of prenatal cases [4], [11], [12]. Langston proposed a “bronchial atresia sequence,” in which the degree of bronchial obstruction, level of obstruction, and timing would result in different patterns of malformation. Langston uses a more descriptive approach to label these lesions. For example, in Langston “classification”, large cyst type and small cyst type/bronchial atresia correspond to CPAM Stocker type I and type II, respectively. CPAM Stocker type II (small cyst type) is likely secondary to bronchial atresia with obstruction. Historically CPAM type I lesion was the most common, accounting for 60 %–70 % in Stocker's defining case series. Recent case series reports [2], [3] showed CPAM type II lesions representing a major subtype. We also experience that CPAM type II is the most common type of CCLL in our practice.

PS occurs in 1:20,000–35,000 individuals and accounts for 0.15 % to 6.40 % of all congenital pulmonary malformations [1]. The anomalous celiac systemic arterial and branches we observed in this case were elastic arterials imitating pulmonary artery [1], and supplied ILS in the right lower lobe [13]. Mild hypertensive-like change in the systemic artery can occur and may become more severe with increasing postnatal age [11], and could be related to the severity of inflammation within the sequestration. The morphological feature of PS is so diverse that effective communication using the term of PS is not possible. PS may be featured with a normal lung tissue, a nonspecific maldeveloped lung tissue, or a spectrum of bronchial atresia sequence, CPAM, and congenital alveolar emphysema [11]. The “hybrid” cases suggest a similar embryological origin for CPAM/CCAM and PS [4]. The most often described combination of “hybrid lesion” is a lesion of ILS and CPAM type 2 [2], [7]. The hybrid lesion has been used clinically to emphasize the importance of seeking an anomalous blood supply in patients who have congenital lung lesions. Although “hybrid lesion” is a well-accepted term in clinical and radiology field, this term may not be popularly accepted in pathological diagnosis [11], [13]. CCLL can be complex when more morphological lesions in the spectrum of bronchial atresia sequence are present. Under this condition, “Hybrid lesion” is more relevant and effective for clinicians and pathologists to understand the overlapping of both imaging and pathological findings, and to avoid missing more lesions during examination. We use hybrid lesion rather than simplified ILS to report this case. It aims to efficiently correlate the terminology and nomenclature applied in the literature currently for multidisciplinary communication.

According to Stocker's criteria, there are overlapping features between CPAM type I and type II, such as both subtypes may have small (<2 cm) or larger cysts (>2 cm), and type II may also have mucous cells. The size of the cysts is not specific for the subtyping of CPAM. Therefore, the precise subclassification of CPAM is challenging in some of the cases, especially in our case complicated with pulmonary infection triggered by SARS-CoV-2 infection. The presence of extremely large cyst (9 cm), clusters of mucous cells (Fig. 2C) and epithelium with papillary projection (Fig. 2D) and rare focus of larger cysts with connection to the dilated alveolar-like spaces (Fig. 2E) were common features of CPAM type I. Bronchiolar epithelium-lined small cysts with or without smooth muscle fibers intermingled with alveolar-like spaces (Fig. 2F) were common features of CPAM type II. The two large cysts could also be cystic changes of the small cysts due to infection. One small case series [13] described 4 cases prenatally diagnosed ILS, which had large cysts and small cysts of CPAM. Langston states that at least 25 % of large cyst type in case series has been reported to have an associated systemic arterial supply [11]. It was unclear what the subtypes of CPAM by Stocker's criterion in the cases of PS with large and small cysts were, since no other features were provided in those reported prenatal hybrid cases [11]. However, a recent large case series [2] showed no case of CPAM type I was associated with a systemic arterial supply.

Bronchial atresia is a well-accepted hypothesis of pathogenetic mechanism in the majority of CCLL [4], [11], [12], particularly in CPAM type II, pulmonary sequestrations and associated lesions, and lobar emphysema. There is currently no known clinical relevance to these distinctions, and many are signed out descriptively in many institutions [2]. Bronchial atresia could be identified in a few case series of CCLL when detailed microdissection was performed [10]. However, microdissection to identify evidence of atretic bronchus is not practical in a busy clinical setting [2]. There are some features that could indicate bronchial atresia/airway obstruction, such as mucous stasis or mucous plugs and muciphages in the bronchus [12], [14]. While the Langston-preferred descriptive approach may help to better understand the pathogenesis, it has potential for miscommunication between pathologist and clinician. Currently, it seems the modified Stocker's classification is still popularly used in clinical practice, although some pathologists use the descriptive approach in combination with Stocker's classification and Langston's bronchial atresia sequence. We were not able to identify bronchial atresia in the specimen of this case, which could be missed during the dissection. Scant mucin mixed with muciphages and acute inflammatory cells in the hilar bronchus and one terminal bronchiole were most likely a secondary event and were insufficient evidence for the airway obstruction associated with this congenital hybrid lesion. The hallmark of COVID-19 infection, such as diffuse alveolar damage and pulmonary artery thrombosis were not identified. Acute and chronic bronchopneumonia, abscesses formation and fibrosis were superimposed with bacterial infection that is commonly seen in late or resolving stage of COVID-19 infection or PS.

Risk of malignancy in CCLL remains poorly defined [3]. Clusters of mucous cells either lining the large cysts or in the surrounding parenchyma were originally reported in 32 % of CPAM type I and were rarely seen in type II (small cyst type) [9]. Although the mucigenic epithelium resembles gastric mucinous epithelium and highlights the embryologic origin of the lung as a foregut derivative, bronchioloalveolar carcinoma has been reported in young adults and children with CPAM type I and bronchogenic cyst [2], [3]. It is presumably related to mucous cell with malignant transformation. Recently one case of CPAM type I with small focus of bronchioalveolar carcinoma was reported and diagnosed incidentally during COVID screening [8]. In our case, there was no cytological or structural atypia to indicate mucous cell with malignant transformation in the cysts of CPAM; there was no features of CPAM type IV or pleuropulmonary blastoma. In one recent study of long-term follow-up, the patients with PS including the cases with epithelium dysplasia and malignant transformation showed 91 %–100 % of patients had no recurrent pneumonia or malignancy after surgical resection of the lesions [15]. The patient has fully recovered following RLL resection. Despite the fact that COVID-19 is ruinously affect people's life and health, it confidently impacted this teenage girl's life with the significance of early uncovering congenital cystic lung disease to prevent recurrent infection or malignant transformation.

The hypothesis of airway obstruction in utero as the common origin for most congenital lung anomalies was based on case series retrospective studies for histopathological examination. The pathogenetic and molecular mechanism of the cystic lung disease remained unclear and controversial. More data from large-scale molecular studies would be warranted to understand the molecular pathogenetic mechanisms of these lesions, specifically for CPAM, PS, and bronchia atresia, and to further develop a uniform classification.

## Provenance and peer review

Not commissioned, externally peer-reviewed.

## Sources of funding

None.

## Ethical approval

A single case study is exempt from ethical approval.

## Consent

Written informed consent was obtained from the patient for publication of this case report and accompanying images. A copy of the written consent is available for review by the Editor-in-Chief of this journal on request.

## Author contribution

Dehua Wang, MD contributed to case report design, data collection, data analysis and writing the paper.

William B. Wheeler contributed to case report design, clinical data collection and writing, data analysis and editing the manuscript.

## Registration of research studies

N/A.

## Guarantor

Dehua Wang.

## Declaration of competing interest

The authors have no conflicts of interest.
